# Reappearance of Chikungunya, Formerly Called Dengue, in the Americas

**DOI:** 10.3201/eid2104.141723

**Published:** 2015-04

**Authors:** Scott B. Halstead

**Affiliations:** Uniformed Services University of the Health Sciences, Bethesda, Maryland, USA

**Keywords:** dengue, chikungunya, arthropod-borne viruses, arthropodborne viruses, flavivirus, togavirus, history of medicine, viruses, zoonoses, mosquitoborne, vectorborne, *Aedes aegypti*, mosquitoes, *Aedes albopictus*, Africa, the Americas, American tropics

## Abstract

After an absence of ≈200 years, chikungunya returned to the American tropics in 2013. The virus is maintained in a complex African zoonotic cycle but escapes into an urban cycle at 40- to 50-year intervals, causing global pandemics. In 1823, classical chikungunya, a viral exanthem in humans, occurred on Zanzibar, and in 1827, it arrived in the Caribbean and spread to North and South America. In Zanzibar, the disease was known as kidenga pepo, Swahili for a sudden cramp-like seizure caused by an evil spirit; in Cuba, it was known as dengue, a Spanish homonym of denga. During the eighteenth century, dengue (present-day chikungunya) was distinguished from breakbone fever (present-day dengue), another febrile exanthem. In the twentieth century, experiments resulted in the recovery and naming of present-day dengue viruses. In 1952, chikungunya virus was recovered during an outbreak in Tanzania, but by then, the virus had lost its original name to present-day dengue viruses.

## Abstract 2

The syndrome was renamed in the twentieth century after it was shown to differ from dengue.

Chikungunya has returned to the Americas after an absence of ≈200 years. The return of this viral exanthem was first recognized on St. Martin, in the Caribbean, in December 2013, and as of January 9, 2015, the US Centers for Disease Control and Prevention reported that the disease had been identified in 42 countries or territories in the Caribbean, Central America, South America, and North America. A total of 1,094,661 suspected and 26,606 laboratory-confirmed chikungunya cases have been reported (http://www.cdc.gov/chikungunya/geo/americas.html). The return of chikungunya virus to the Americas provides an opportunity to revisit the epidemiology of this zoonotic togavirus from Africa and to contrast it with that of dengue viruses, flaviviruses that are maintained as zoonoses in Southeast Asia. All of these viruses can be transmitted by *Aedes aegypti* and *Ae. albopictus* mosquitoes in an urban cycle. In the course of history, a remarkable name change has taken place because of the similarities between the clinical syndromes caused by dengue and chikungunya virus infections. The story of how the term dengue was originally applied to what we now call chikungunya and then subsequently applied to what we now call dengue should be well known by persons who deal with these 2 similar, but crucially different, global diseases. For more details on the name switch, the reader should consult the historical account by Carey (*1*).

## The Chikungunya Epidemic of 1827–1828

According to a contemporary medical observer of the chikungunya epidemic of 1827–1828, S. Henry Dickson, Professor of Medicine, Medical College of South Carolina,

“[A]n arthritic fever with cutaneous exanthema [(*2*)]… appeared first in the island of St. Thomas, the chief town of which it invaded in September, 1827, attacking in rapid succession almost every individual in a population of about 12,000. Towards the end of October, it passed over to the neighbouring island of St. Croix. We hear of it, in November, in St. Bartholomew’s, and in Antigua in January, 1828. It prevailed at Havanna [sic] in the succeeding April, at New Orleans in May and June; and in July and August affected very generally the inhabitants of Charleston, South Carolina, and reached Savannah (Georgia) in September and October.” (*3*)

On clinical evidence, this outbreak was caused by chikungunya virus. However, that clinical evidence is supplemented by the eyewitness report and the epidemiologic detective work of James Christie, physician to His Excellency Syud Bargash, Sultan of Zanzibar, 1865–1873. In his report, published in the British Medical Journal in 1872, Christie described the onset in July 1870 on Zanzibar of an acute febrile exanthem that rapidly achieved epidemic proportions (*4*). He himself was sick and in early convalescence experienced “pain on rising from my chair [that] was very severe after a short interval of rest....I suffered severely [from joint pain] for more than two months afterward” (*4*). From older patients in his practice, Christie learned that there had been a similar epidemic on Zanzibar 48 years earlier that was known by the Swahili term kidinga pepo (also called kidenga or kidyenga pepo). In this phrase, “ki… simply means ‘a kind of,’” the word “dinga or dyenga… means sudden cramp-like seizure,” and “pepo, means wind and also a spirit… so that the full designation of the term signifies a disease characterized by a sudden cramp-like seizure, caused by an evil spirit” (*5*).

## Origin of the Term Dengue

Christie explicitly linked the 1827–1828 epidemic of kidinga pepo in the Americas to the 1823 epidemic on Zanzibar. He noted that published reports indicated that the 1823 epidemic soon spread from Zanzibar to Gujarat, India, and then to Calcutta, India, and by 1824 it had spread to Rangoon in present-day Myanmar. In 1827, there were reports of a similar disease in St. Thomas in the West Indies. Christie stated, “I am of the opinion that both the disease and its designation were imported in the West Indian Islands direct from the East Coast of Africa” (*5*). It was in the West Indies, as Christie observed, that the medical term dengue was introduced. Dumaresq, an observer of the dengue epidemic in New Orleans, Louisiana, USA, in 1828 commented, “The disease alluded to is supposed to have been brought from Africa, with some slaves imported into the Havana. In that place it obtained the name of Dingee, Dengue, Danga, etc. It was there very prevalent, and also in Barbadoes [sic], where it received the name of Dandy fever, from the stiffened form and dread of motion in patients” (*6*). In New Orleans, the disease “spread was so rapid among the inhabitants that in eight or ten days at least one third of the population was laboring under its influence, including persons of all ages and different sexes” (*6*). Dumaresq goes on to say,

“A person on the disappearance of this fever would attempt to rise from bed, feeling not much loss of strength, and a consciousness of being able to move about and attend to a little to business; but how egregiously would he be mistaken when he assumed the upright posture! The joints felt as if fettered or anchylosed, and the advance of one foot or leg beyond the other, would cost more pain and effort than the purpose for which it may have been advanced was worth, —aye,—a thousand times told!” (*6*)

The arthritic component of this febrile exanthem is unique to epidemic human chikungunya infections. It has been variously called scarlatina rheumatica, exanthesis arthrosia, and an eruptive articular or rheumatic fever (*7*).

An interesting further insight into the colloquial Spanish meaning of dengue may contribute to an understanding why this term prevailed so quickly. In 1952, when Sabin inquired into the etymology of the term dengue, the standard Spanish dictionary meaning was affectation (*8*). Dengue researchers at that time were unable to make a connection between this term and characteristic signs and symptoms of dengue. However, an interesting connection does exist, but it is to the disease caused by what we now call chikungunya, not dengue. In 1828, contemporary observers were struck by the post-illness arthralgia and disability caused by dengue (i.e., present-day chikungunya), including the post-illness symptoms cited above in comments by Dumaresq (*6*). Stedman noted an even more extreme manifestation of dengue, reporting, “It is even said that when the disease first appeared in St. Thomas, several negroes, who, being all at once attacked with pain in the knees, had fallen down, were actually apprehended by the police for drunkenness” (*9*). Lehman, the lazaretto (i.e., quarantine station) physician for the port of Philadelphia, Pennsylvania, USA, interviewed a ship captain from Cuba who declared that, “It [dengue] is a vulgar phrase, and implies a ‘staggering weakness,’ and is somewhat similar in its import to our term of ‘corned’ [drunk] as applied to a man reeling about from intoxication” (*10*). The original meaning of kidinga pepo has been consistently maintained from Swahili to the colloquial eighteenth century Spanish term dengue as an apt name for a disease that produces a post-illness stagger.

## Discovery of Chikungunya Pandemics

When Dr. Christie left Africa in 1876 to assume a post as a lecturer on public health at Anderson’s College, Glasgow, he discovered reports in the medical literature of 3 pandemics of kidinga pepo. The epidemic of 1870–1880 had begun in Zanzibar and then spread to India and Southeast Asia. That epidemic had been preceded by one in 1823–1828 that originated in Africa and then spread to India, Southeast Asia, and the Americas, and that epidemic had been preceded by an even earlier one in 1779–1785 that was reported in Egypt, Africa, Arabia, India, and Southeast Asia (*5*). Of interest to contemporary observers, in 1872, an epidemic of kidinga pepo affected most inhabitants of low-lying areas of Réunion Island, the site where the chikungunya pandemic of 2005–2006 was first recognized (*11*). Dr. Christie suspected that the illness in all 3 pandemics was kidinga pepo because he had personally observed the 1870 epidemic spread from Zanzibar to the entire Indian subcontinent and progress on to Southeast Asia. Then, from a published report, he learned of an epidemic of kidinga pepo in Cairo in 1779. This report was followed by others that reported outbreaks in Arabia, India, and Southeast Asia. This epidemic reached Indonesia in 1779, where another astute physician, David Bylon, municipal surgeon for the city of Batavia (now Jakarta, Indonesia), acquired the disease. Dr. Bylon described the epidemic in a classic account, which has been widely cited as the initial clinical description of dengue fever:

“It was last May 25, in the afternoon at 5:00 when I noted while talking with two good friends of mine, a growing pain in my right hand, and the joints of the lower arm, which step by step proceeded upward to the shoulder and then continued onto all my limbs; so much so that at 9:00 that same evening I was already in my bed with a high fever.… It has now been three weeks since I… was stricken by the illness, and because of that had to stay home for 5 days; but even until today I have continuously pain and stiffness in the joints of both feet, with swelling of both ankles; so much so, that when I get up in the morning, or have sat up for a while and start to move again, I cannot do so very well and going up and down stairs is very painful.” (*12*, as translated by K. DeHeer)

Carey, who rediscovered Christie’s work, noted that chikungunya pandemics originating in eastern Africa had crossed the Indian Ocean at roughly 40- to 50-year intervals: the 1770s, 1824, 1871, 1902, 1923, and 1963–1964 (*1*). To those cycles we now can add 2005–2014. The last 2 trans–Indian Ocean pandemics occurred in the modern virologic era and have been documented by the isolation of virus. In 1963–1964, a chikungunya epidemic swept down the eastern coast of India from Calcutta to Sri Lanka (*13*–*15*). It was this epidemic that resulted in the recognition of the pronounced clinical differences between syndromes caused by dengue viruses and chikungunya virus. During the 1964 epidemic in Vellore, in southern India, most of the 275 patients with virologically or serologically confirmed chikungunya were adults (*16*). The patients had “sudden onset… of fever, headache and severe pains in the joints, these last being the dominant complaint. The pains mainly affected the small joints of the hands, wrist and feet, but frequently occurred in the knees as well” (*16*). After 1964, chikungunya virus gradually disappeared from India, with the last isolates recorded in 1972 (*17*,*18*). During 2005–2006, a chikungunya epidemic that originated in eastern Africa was observed on Réunion Island and then in Mauritius, Madagascar, Mayotte, and Seychelles (*19*); the epidemic soon spread to India and Southeast Asia (*20*,*21*). The Réunion Island outbreak was noteworthy because *Ae. albopictus* mosquitoes were efficient vectors that were aided by a genetic mutation in the virus (*22*). This virus was subsequently introduced into Europe by tourists returning from visits to Réunion Island or India, resulting in modest outbreaks of autochthonous *Ae. albopictus* mosquitoborne chikungunya in southeastern France and northeastern Italy (*23*–*24*).

## History of Disease Caused by Dengue Viruses

The first clinical description of a syndrome likely to have been caused by a dengue virus was one by Benjamin Rush, who in 1789 described an epidemic of a disease he called bilious remitting fever (*25*). The epidemic occurred from mid-August through September 1780 in Philadelphia, principally among residents living along the Delaware River waterfront. According to Rush (*25*):

“The fever generally came on with rigor… In some persons it was introduced by a slight sore throat…. The pains which accompanied this fever were exquisitely severe in the head, back and limbs. The pains in the head were sometimes in the back parts of it and sometime occupied only the eyeballs…. A few complained of their flesh being sore to touch… the disease was sometimes believed to be a rheumatism. But, its more general name among all classes of people was *breakbone fever*…. A nausea universally, and in some instances, vomiting, accompanied by a disagreeable taste in the mouth, accompanied this fever…. A rash often appeared on the third and fourth days.”

Rush’s description of bilious remitting fever was well known to physicians who attended to patients during the 1828 outbreak in the Caribbean. However, at the same time, George Stedman, a former president of the Royal Medical Society of Scotland, who practiced medicine on St. Croix, felt that the 1828 dengue was quite different from bilious remitting fever. He observed, “I think that it will be evident to everyone who pays the least attention to the symptoms, that the diseases, though somewhat alike in a few symptoms, are essentially different” (*9*). The principal distinctions made by Stedman were in the suddenness of the onset and the nature and duration of the after-pains of dengue (present-day chikungunya) (*9*). Christie also recognized the existence of 2 distinct febrile exanthems, 1 with and 1 without post-illness arthritis. He cited a description of dengue “with an almost entire absence of the articular pains”; this description of illness during an 1853–1854 epidemic in Calcutta was from The Science and Practice of Medicine (*26*), an authoritative text authored by William Aitken (*4*,*27*).

## History of Chikungunya Name Change

Why then did chikungunya lose and dengue gain a name? Throughout the nineteenth century, astute observers of outbreaks in the Americas and India recognized the clinical differences between dengue and breakbone fever, principally the duration of fever and the occurrence of post-illness arthritis (*27*–*29*). The term dengue was in use to describe an epidemic that reached India in 1871 from Zanzibar and eastern Africa (*30*–*32*). However, once Reed and co-workers identified *Ae. aegypti* mosquitoes as the vector of yellow fever, the epidemiologic similarities between dengue and yellow fever led researchers in Lebanon, Australia, and the Philippines to investigate the etiology of dengue and the mode of transmission of dengue virus (*33*–*36*). At that time, by coincidence, dengue but not chikungunya viruses were endemic at these 3 sites. Two groups, one in Australia and the other in the Philippines, apparently successfully transmitted virus from sick humans to healthy volunteers through the bite of infected *Ae. aegypti* mosquitoes (*35*) and *Culex fatigans* (now called *C. quinquefasciatus*) mosquitoes (*36*). Ashburn and Craig successfully infected human volunteers by inoculating them with diatomaceous earth–filtered blood from patients with dengue, thereby proving a viral etiology for the disease (*36*). It remained for Siler and Simmons and co-workers in the Philippines in 1923 and 1929 to definitively demonstrate that *Ae. aegypti* mosquitoes, but not *C. quinquefasciatus* mosquitoes, are a biological vector of dengue virus (*37*–39). During the first half of the twentieth century, many experimental infections with dengue viruses were studied in human volunteers, and the clinical features of the infections were recorded in detail; all authors referred to the disease under study as dengue. In 1952, decades after these experiments were begun, a virus was recovered from an outbreak of an exanthematous febrile disease in Southern Province, Tanganyika Territory (now in Tanzania). The virus was called chikungunya, which in the Makonde language (spoken by an ethnic group in southeast Tanzania and northern Mozambique) means that which bends up (*40*). The name change was complete.
